# Factors Affecting Thyroid Volume in Children Aged 4 to 18 Years

**DOI:** 10.3390/diagnostics15151980

**Published:** 2025-08-07

**Authors:** Nevena Folić, Marko Folić, Miloš N. Milosavljević, Ana V. Pejčić, Slobodan Janković, Maja Vulović, Milos Stepovic, Isidora Mihajlović, Jovana Milosavljević

**Affiliations:** 1 Department of Pediatrics, Faculty of Medical Sciences, University of Kragujevac, 34000 Kragujevac, Serbia; nevena.folic@yahoo.com (N.F.); dora_mihajlovic@hotmail.com (I.M.); 2 Pediatric Clinic, University Clinical Center Kragujevac, 34000 Kragujevac, Serbia; 3 Center for Pharmaceutical and Pharmacological Research, Faculty of Medical Sciences, University of Kragujevac, 34000 Kragujevac, Serbia; markof@fmn.kg.ac.rs; 4 Department of Clinical Pharmacology, University Clinical Center Kragujevac, 34000 Kragujevac, Serbia; slobodan.jankovic@medf.kg.ac.rs; 5 Department of Pharmacology and Toxicology, Faculty of Medical Sciences, University of Kragujevac, 34000 Kragujevac, Serbia; milosavljevicmilos91@gmail.com (M.N.M.); anapejcic201502@yahoo.com (A.V.P.); 6 Department of Anatomy, Faculty of Medical Sciences, University of Kragujevac, 34000 Kragujevac, Serbia; maja@fmn.kg.ac.rs (M.V.); jowana.ilic@yahoo.com (J.M.)

**Keywords:** thyroid gland, thyroid volume, children, ultrasound

## Abstract

Background and Objectives: Ultrasound measurement of thyroid volume is not routinely performed in children without suspected thyroid disorders. However, pediatricians must be aware of the normal thyroid volume values in children in order to recognize and treat thyroid disorders in children on time. Therefore, this study aimed to explore factors that influence thyroid volume in children aged 4 to 18 years and to provide descriptive data on thyroid volume across this age range. Materials and Methods: This was a prospective, non-interventional cross-sectional study conducted on a population of children aged 4 to 18 years without confirmed thyroid disorders. We used validated formulas for calculating thyroid volume that integrate the linear dimensions of this organ, such as length, width, and depth, as well as the appropriate correction factor. The Spearman or Pearson correlation coefficient was calculated to assess the relationship between total thyroid volume and various continuous variables, while multiple linear regression analysis was used to evaluate the effect of potential predictors on the total thyroid volume. Results: The study included 100 children, predominantly girls (75.0%). Significant positive correlations with thyroid volume were found for age, height, weight, body mass index (BMI), body surface area, serum creatinine, birthweight, and number of comorbidities, while calcitonin was negatively correlated with children’s thyroid volume. We identified age, BMI, and serum creatinine as significant independent positive predictors of thyroid volume in children. Conclusions: Age, BMI, and serum creatinine emerged as significant independent positive predictors of thyroid volume and should be considered when interpreting pediatric thyroid ultrasound measurements.

## 1. Introduction

The name of the thyroid gland comes from the Greek word thyreoeidos (thyreos—shield; eidos—from). Thyroid development begins by the third week of gestation and is completed by the eleventh week [1]. The thyroid gland consists of two lateral lobes that are connected by a constriction (isthmus) located in front of the trachea and weighs about 15–25 g in adults [2]. The lobes of the thyroid gland measure about 40 mm in height, 15–20 mm in width, and 20–39 mm in thickness [3]. The dimensions can be drastically changed due to diseases. The gland is covered by a thin fibrous capsule without true lobulations. The inner layer of capsule extends into the thyroid gland, creating septa that separate the glandular tissue into tiny lobules. Meanwhile, the outer layer is continuous with the pretracheal fascia (middle layer of the cervical fascia) and connects the gland to the cricoid and thyroid cartilages through a thickened portion of fascia, forming the posterior suspensory ligament of the thyroid gland, also referred to as Berry’s ligament, which is responsible for the elevation of the thyroid gland during swallowing [4]. The lateral lobes of the thyroid gland are located in relation to the trachea, larynx, pharynx, and esophagus medially, and boarded by the carotid sheath laterally [1]. Anteriorly, the gland is covered by the superficial fascia and platysma, and posteriorly to each lobe are located the parathyroid glands [2]. The Zuckerkandl tubercle is a posterolateral projection of the lateral thyroid lobe and may be confused with a neoplasm or another mass, and it represents an important landmark in thyroid surgery [5].

Accurate assessment of thyroid volume, in patients of all ages, is important for the correct diagnosis of goiter and for the ultrasound monitoring of thyroid disease [6]. This is particularly important in pediatrics because linear measurements of the developing thyroid gland do not correlate well with age, gender, or variation in body composition [6]. Various methods, such as ultrasound, visual inspection, and palpation, are used to assess thyroid size and volume [6]. Although thyroid size can be easily assessed by physical examination, due to the superficial location of the gland, palpation has low sensitivity and specificity for the management and diagnosis of thyroid disorders [6]. Therefore, thyroid volume measured by ultrasound is a more accurate method than physical examination. Inaccurate calculation of thyroid size and volume may lead to false-positive or false-negative diagnoses of thyromegaly, which may lead to unnecessary or delayed care [6].

In 1997, the World Health Organization published a nomogram with predicted values for thyroid volume in boys and girls aged 6 to 15 years. Data on pediatric development and thyroid volume from different parts of the world correlate well with the WHO nomograms [7]. A study of the thyroid gland in children and adolescents from a region in Ukraine showed that the upper limit of normal thyroid volume for 13-year-old girls is 12.1 mL, which roughly corresponds to the WHO data of 13.1 mL [8]. In Japan, the upper limit of normal thyroid volume for 13-year-old girls is 11.9 mL, while in an iodine-sufficient area in Switzerland, it is approximately 12.5 mL [7].

Bernardes et al. developed nomograms of fetal thyroid volume, fetal thyroid area, and fetal thyroid transverse diameter during gestation to identify thyroid dysfunction promptly [9]. Funaki et al. developed a nomogram of fetal thyroid volume using two-dimensional ultrasound and determined the normal period of appearance of fetal distal femoral and proximal tibial ossification centers for assessing fetal thyroid function [10]. Some studies have examined the effects of socioeconomic status and parental education on the time from diagnosis to biopsy in children with thyroid nodules. There were no gender or racial differences in the odds of malignant neoplasm of the nodule, although female gender, older age, and white race were associated with a higher odds of receiving a biopsy, while higher parental education significantly influenced a shorter time from diagnosis to biopsy [11].

Although detailed and precise, the WHO nomogram has its shortcomings. Certainly, the most important shortcoming of this nomogram is that it does not cover children under the age of 6 years. Furthermore, despite the availability of WHO thyroid volume reference values for children aged 6 to 15 years, there remains a lack of detailed information on how various clinical, anthropometric, and biochemical factors affect thyroid volume throughout childhood and adolescence. Therefore, this study aimed to examine those factors in a cohort of Serbian children aged 4 to 18 years and to provide percentile distributions of thyroid volume for descriptive purposes.

## 2. Materials and Methods

### 2.1. Study Design

This was a prospective, non-interventional cross-sectional study. The Ethics Committee of the University Clinical Center Kragujevac approved the conduct of our research on 23 May 2024 (Decision Number 01/24-276).

### 2.2. Study Population

The study population consisted of children aged 4 to 18 years who came for an outpatient examination or were hospitalized at the Pediatric Clinic of the University Clinical Center Kragujevac, Serbia, due to various etiologies. In all children included in the study, according to the opinion of pediatricians who did not participate in the study, there was a need for an ultrasound examination of the thyroid gland. Children were included in the study only after their parents or guardians signed a voluntary written consent. Parents or guardians were previously informed in detail about the objectives of the study and its significance, as well as the procedures that would be performed on their children. The inclusion criteria for the participation of children in the study were being aged from 4 to 18 years, clinically and ultrasound-confirmed absence of any anatomical or functional disorder of the thyroid gland, normal somatic growth and development of the child, normal cognitive development of the child, and written consent for participation in the study signed by the parent or guardian. On the other hand, the study excluded children under 4 years of age, those over 18 years of age, children with ultrasound and/or clinically verified anatomical and/or functional thyroid disorders, children exhibiting growth retardation, children with cognitive developmental disorders, and children for whom the requisite anthropometric, clinical, and sociodemographic data could not be obtained.

We used a “convenient” consecutive sample when recruiting children.

### 2.3. Study Variables

The main dependent variable in this study was thyroid gland volume. The volume of each thyroid lobe was calculated using the following formula:

Volume (mL) = Length (cm) × anterior-posterior diameter (cm) × Width (cm) × Correction Factor. Two different correction factors were used for the calculations: 0.523 (denoted as Formula 1) [8] and 0.479 (denoted as Formula 2) [12].

The total thyroid volume was then calculated as the sum of the volumes of both lobes. Image evaluation and morphometric measurements were performed by experts in the area of pediatrics (N.F. and I.M.). Two independent observers who made the measurement were blind to the protocol and showed high inter-rater reliability (Pearson’s r above 0.90).

In addition to thyroid volume, the following data were collected: demographic information such as age, gender, and place of residence; anthropometric data such as height, weight, body mass index (BMI), and body surface area (BSA); medical history related to presence of comorbidities and use of medications; laboratory parameters such as thyroid-stimulating hormone (TSH), free thyroxine (FT4), free triiodothyronine (FT3), anti-thyroid peroxidase antibodies, thyroglobulin, calcitonin, parathormone, red blood cell count, white blood cell count, platelet count, hemoglobin, hematocrit, blood glucose, urea, serum creatinine, alanine aminotransferase (ALT), and aspartate aminotransferase (AST); perinatal data, such as the type of delivery, gestational week at birth, birthweight, Apgar score at birth, and birth order; maternal characteristics, such as smoking status, alcohol consumption, and history of chronic disease; paternal characteristics, including smoking and alcohol consumption; and family history of thyroid disease. BMI was calculated using the formula BMI = weight (kg)/[height (cm)^2^], while BSA was calculated using the formula BSA = weight^0.425^ (kg) × height^0.725^ (cm) × 0.007184 [13].

Height-for-age and weight-for-age percentiles were calculated for each participant using the Centers for Disease Control and Prevention (CDC) growth charts for the ages of 2–20 years, adjusted for age and sex [14]. BMI-for-age percentiles were calculated using the CDC Child and Teen BMI Calculator, accounting for age and sex, and participants were classified into following BMI categories: underweight (<5th percentile), healthy weight (5th–84th percentile), overweight (85th–94th percentile), and obesity (≥95th percentile) [15,16].

### 2.4. Data Analysis

Statistical analyses were performed using the Statistical Package for the Social Sciences (SPSS), version 18. Descriptive statistics were used to summarize the data. For numeric variables, we reported the mean, median, standard deviation (SD), and range; categorical variables were summarized using frequencies and percentages. Percentiles of the right and left thyroid lobe volumes, as well as total thyroid volume, were calculated for the study population across three age groups: 4–10 years, 11–14 years, and 15–18 years. Spearman or Pearson correlation coefficient was calculated to assess the relationship between total thyroid volume and other continuous variables, based on the results of normality tests (Kolmogorov–Smirnov and Shapiro–Wilk tests). To evaluate the effect of potential predictors on total thyroid volume (as the outcome variable), we conducted multiple linear regression analysis using backward elimination with a probability of F to remove a predictor of ≤0.1. The validity of the regression model was assessed through analysis of variance using the F-statistic, and the proportion of variability explained was indicated by the R^2^ value. The impact of individual predictors on the outcome was evaluated using their regression coefficients (B) and corresponding 95% confidence intervals (CIs). A *p*-value of < 0.05 was considered statistically significant.

## 3. Results

The study included 100 participants aged 4 to 18 years (mean ± SD: 12.51 ± 3.61 years), with a predominance of females (75.0%). Comorbidities were present in 49 participants (49.0%), with an average of 0.51 ± 0.63 comorbidities per individual (median: 0; range: 0–2). The most frequently reported comorbidities were diabetes and asthma, each affecting 10 individuals (10.0%), followed by allergies and insulin resistance (8.0% each) and obesity (6.0%). Medication use was reported by 34 participants (34.0%). Among the participants, 12 (12.0%) were using oral antidiabetic medications, 9 (9.0%) were receiving insulin therapy, and 6 (6.0%) were treated with montelukast. A chronic disease was reported by 38 mothers (38.0%). Smoking was reported in 45 mothers (45.0%) and 46 fathers (46.0%). Alcohol consumption was uncommon among mothers, with only 1 (1.0%) reporting alcohol use, while 14 fathers (14.0%) reported alcohol consumption. A family history of thyroid disease was present in 41 participants (41.0%). Additional descriptive characteristics are presented in Table 1.

**Figure 1:**
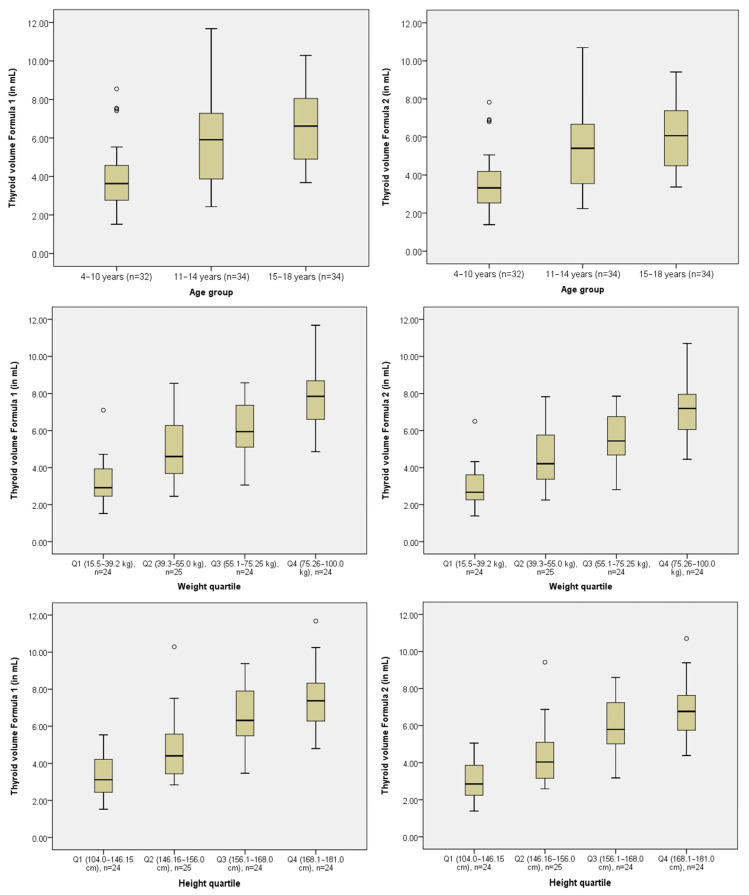
Box plots of thyroid volume calculated using Formula 1 and Formula 2 stratified by age group, weight, and height quartiles.

The mean total thyroid volume calculated using data obtained with Formula 1 was 5.51 ± 2.22 mL and 5.05 ± 2.03 mL using Formula 2. Percentiles of the right and left thyroid lobe volumes, as well as the total thyroid volume, were calculated across three age groups (4–10, 11–14, and 15–18 years) using both formulas (Table 2). As expected, volumes increased progressively with age. For example, the median (50th percentile) total thyroid volume for the 4–10 year age group was 3.63 mL using Formula 1 and 3.325 mL using Formula 2. In the 15–18 year group, median volumes reached 6.62 mL and 6.07 mL. Figure 1 displays box plots of thyroid volume calculated by both formulas, stratified by age group, weight, and height quartiles.

Table 3 presents the statistically significant correlation coefficients between total thyroid volume and various numerical variables, both in the total sample and within specific age groups. Significant positive correlations with total thyroid volume were found for age, height, weight, BMI, BSA, serum creatinine, birthweight, and number of comorbidities in at least one age group. Negative correlation was observed for calcitonin. The correlation between parathormone levels and total thyroid volume varied across age groups, with a significant positive correlation observed in the 4–10 years group and a significant negative correlation found in the 15–18 years group, while no significant correlations were detected in the 11–14 years group.

Multiple linear regression analysis using backward elimination identified age, BMI, and serum creatinine as significant independent positive predictors of total thyroid volume for both formulas (Table 4). Both models explained 55.1% of the variance in total thyroid volume (R^2^ = 0.551, F = 37.592, *p* < 0.001 for Formula 1 and R^2^ = 0.551, F = 37.611, *p* < 0.001 for Formula 2).

## 4. Discussion

The results of our study indicate that there is a constant increase in the volume of the thyroid gland during the period of childhood. We observed that the volume of the thyroid gland in children is positively correlated with age, values of the most important anthropometric parameters, serum creatinine concentration, birthweight, and the number of comorbidities. On the other hand, the volume of the thyroid gland in children negatively correlates with calcitonin and FT3. Finally, we showed that age, BMI, and serum creatinine concentration are the most important predictors of thyroid volume in childhood. These findings relate mostly to children older than 6 years, since only a few children from the study sample were 4–6 years old.

Ultrasonography of the thyroid gland is not a diagnostic method that is routinely performed in children [17]. Moreover, it is recommended in situations where there is suspicion of thyroid disease in children, such as congenital hypothyroidism, thyroid cancer, iodine deficiency, and other forms of thyroid function and/or structure disorders [17]. There are objective difficulties when performing ultrasonography of the thyroid gland in children and interpreting the results [18]. First of all, often, there is a problem with the lack of cooperation among children during the implementation of this procedure [18]. Also, the short neck of children often significantly complicates the accurate measurement of the length of the thyroid gland [18]. However, thyroid ultrasonography is a non-invasive, rapid, and safe diagnostic method that is widely used for the early diagnosis of thyroid disease in children [19,20]. A change in the size of the gland is one of the most obvious alterations that occurs in children with thyroid issues [21]. Therefore, in order to identify and treat thyroid disorders in children, we need to know the predicted thyroid gland dimensions in euthyroid children.

Back in 1997, the World Health Organization published a nomogram with predicted thyroid volume values for children aged 6 to 15 years that were adjusted for gender and age [7]. However, the results of a large number of studies have indicated that the volume of the thyroid gland in children is also influenced by parameters such as ethnic affiliation and iodine intake [19,20]. Due to this, nowadays, we have national reference values for thyroid volume in children around the world, such as those in Malaysia [22], Poland [23], Turkey [24], Iran [25], and other countries. However, despite these ethnic differences, the factors that influence thyroid volume in children are generally common in all parts of the world. It is known that all three linear dimensions and the volume of the thyroid gland grow constantly throughout childhood, with a particularly pronounced jump during puberty. The results of numerous studies have confirmed that there is a strong positive correlation between age and thyroid gland volume in children [13,21,26,27]. On the other hand, the relationship between thyroid volume and gender is controversial. We did not show that there is a statistically significant difference in thyroid gland volume between girls and boys of the appropriate age, which is consistent with the results of a large number of other researchers [13,18,20,27,28]. However, Kaba and colleagues showed that girls aged 12–13 years have statistically significantly higher thyroid gland volume than boys of the same age [29]. The WHO nomograms contain different predicted values for thyroid volume for boys and girls of the appropriate age [7], but the difference between these values appears to be negligible.

The results of several studies have shown that anthropometric parameters are positively correlated with thyroid volume in children. We showed that the thyroid volume of children increases with height, weight, BMI, and BSA, which was previously confirmed in the population of children aged 6 to 12 years in China [13]. Zou et al., in their study conducted on a sample of 729 children aged 6 to 12 years in China, also showed that thyroid volume positively correlated with BSA [26]. BSA and BMI are anthropometric indices whose calculation is based on body height and body weight [30]. The results of multiple linear regression in our study identified BMI as an important predictor of thyroid volume in children, which is consistent with the results of the study by de Souza et al. [20]. However, other authors proved by regression analysis that BSA is a significant positive predictor of thyroid volume in children [13,26,31,32].

We observed that there is a positive correlation between serum creatinine concentration and thyroid volume. Also, we seem to be the first to observe that serum creatinine is a positive predictor of thyroid volume in children. The relationship between thyroid anatomy and function and renal function is complex. Thus, it is known that primary hypothyroidism is associated with a reversible increase in serum creatinine concentration in both adults and children [33,34]. It is most likely that, in patients with hypothyroidism, there is a decrease in glomerular filtration rate in the kidneys and a consequent increase in serum creatinine concentration [35]. On the other hand, in patients with hyperthyroidism, an inverse relationship is found between serum creatinine concentration and glomerular filtration rate [36]. The results of a large Korean national study showed that an increased TSH was associated with decreased estimated glomerular filtration rate [37]. Łebkowska et al. observed that there is a positive correlation between thyroid volume and serum creatinine concentration in a population of kidney transplant patients [38]. However, we believe that further analysis of the nature of the relationship between serum creatinine and thyroid volume in children requires prospective studies on a larger sample of children, with more detailed monitoring of renal and thyroid function.

To the best of our knowledge, we are the first to show that there is a negative correlation between calcitonin concentration and thyroid volume in children. It appears that there is a rational explanation for this correlation. On the one hand, it is known that, during childhood, there is a continuous growth in the volume of the thyroid gland [13,21,26,27]. Again, on the other hand, calcitonin concentrations physiologically decline during childhood [39].

We should emphasize the limitations of our study. The first limitation relates to the relatively small sample size we recruited for the purposes of this study. Additionally, the small number of participants in the 4–6 years age range prevented a separate analysis of this group, limiting our ability to draw age-specific conclusions for the youngest children included in the study. Consequently, data for this age range were combined within a larger 4–10 years group. Unicentricity is another important limitation of our study. Finally, we did not consider iodine intake as an important variable that may influence thyroid volume in children. However, thanks to the decades-long implementation of the dietary salt iodization program in the Republic of Serbia, the problem of iodine deficiency is considered to have been completely eliminated [40]. Finally, the distribution of our study population by gender could be a significant limitation of our study, given that girls made up as much as three-quarters of our study population.

## 5. Conclusions

In conclusion, this study identifies age, BMI, and serum creatinine as independent predictors of thyroid volume in children. These findings may assist clinicians in interpreting pediatric thyroid ultrasound measurements, while the provided percentile data offer additional descriptive insight into thyroid volume distribution across age groups.

## Figures and Tables

**Table 1:** Basic characteristics of the study population (*n* = 100).

Characteristics	Mean ± Standard Deviation; MEDIAN (Range) or Number (%)
**Age (years)**	12.51 ± 3.61; 13.00 (4–18)
**Gender**	
Male	25 (25.0%)
Female	75 (75.0%)
**Place of residence**	
Urban	73 (73.0%)
Rural	27 (27.0%)
**Height (cm) (*n* = 97)**	153.87 ± 18.15; 156.00 (104.0; 181.0)
**Weight (kg) (*n* = 97)**	56.46 ± 22.32; 55.00 (15.5; 100.0)
**Body mass index (kg/m^2^) (*n* = 97)**	22.87 ± 5.96; 22.09 (11.76; 37.42)
**Height-for-age percentile (*n* = 97)**	63.4 ± 35.1; 78.2 (0.3; 99.9)
**Weight-for-age percentile (*n* = 97)**	71.3 ± 36.1; 90.2 (0.1; 99.9)
**Body mass index category (*n* = 97)**	
Underweight (<5th percentile)	8 (8.0%)
Healthy weight (5–84th percentile)	35 (35.0%)
Overweight (85–94th percentile)	20 (20.0%)
Obesity (≥95th percentile)	34 (34.0%)
**Body surface area (m^2^) (*n* = 97)**	1.52 ± 0.38; 1.55 (0.67; 2.20)
**Birth order (*n* = 97)**	
1st	50 (50.0%)
2nd	31 (31.0%)
3rd	13 (13.0%)
4th	3 (3.0%)
**Type of delivery (*n* = 97)**	
Vaginal delivery	72 (72.0%)
Cesarean section	25 (25.0%)
**Gestational week at birth (*n* = 94)**	39.00 ± 2.16; 40.00 (30.0; 42.0)
**Birthweight (g) (*n* = 96)**	3256.93 ± 540.23; 3275.0 (1240.0; 5100.0)
**Apgar score at birth (*n* = 88)**	9.01 ± 0.70; 9.00 (5.00; 10.00)
**Laboratory parameters**	
Thyroid-stimulating hormone (TSH) (uIU/mL)	2.11 ± 1.14; 1.90 (0.10; 5.10)
Free thyroxine (FT4) (pmol/L)	15.57 ± 2.34; 15.80 (8.15; 20.80)
Free triiodothyronine (FT3) (pmol/L) (*n* = 99)	4.98 ± 2.69; 4.70 (2.60; 30.00)
Anti-thyroid peroxidase antibodies (IU/mL)	28.43 ± 115.94; 9.05 (−1.00; 1140.00)
Thyroglobulin (ng/mL)	12.84 ± 9.44; 10.60 (0.10; 54.70)
Calcitonin (ng/L)	3.84 ± 4.72; 3.00 (−1.00; 40.00)
Parathormone (pg/mL)	18.96 ± 11.77; 16.90 (4.80; 71.30)
Red blood cell count (10^12^/L)	4.67 ± 0.41; 4.61 (3.75; 6.68)
White blood cell count (10^9^/L)	7.32 ± 2.31; 7.10 (3.95; 19.90)
Platelet count (10^9^/L)	259.97 ± 57.87; 256.00 (138.00; 420.00)
Hemoglobin (g/L)	129.12 ± 16.17; 131.00 (12.40; 152.00)
Hematocrit (L/L)	0.38 ± 0.03; 0.38 (0.26; 0.44)
Blood glucose (mmol/L)	5.87 ± 6.66; 4.60 (3.20; 52.00)
Urea (mmol/L)	4.63 ± 7.17; 3.90 (1.70; 75.00)
Serum creatinine (μmol/L)	54.09 ± 12.95; 53.00 (19.00; 85.00)
Alanine aminotransferase (U/L)	20.67 ± 12.10; 17.00 (8.00; 84.00)
Aspartate aminotransferase (U/L)	24.38 ± 7.56; 23.00 (13.00; 58.00)
**Right lobe volume (mL)**	
Formula 1	2.89 ± 1.25; 2.67 (0.96; 6.43)
Formula 2	2.65 ± 1.15; 2.44 (0.88; 5.89)
**Left lobe volume (mL)**	
Formula 1	2.61 ± 1.07; 2.49 (0.56; 5.25)
Formula 2	2.39 ± 0.98; 2.28 (0.51; 4.81)
**Total thyroid volume (mL)**	
Formula 1	5.51 ± 2.22; 5.37 (1.52; 11.68)
Formula 2	5.05 ± 2.03; 4.92 (1.39; 10.70)

**Table 2:** Percentiles of right and left lobe volumes and total thyroid volume according to age group and formula.

Age Group/Characteristics	Percentile						
	5	10	25	50	75	90	95
**4–10 years (*n* = 32)**							
Right lobe volume (Formula 1)	0.9600	1.1330	1.3350	1.7950	2.3600	3.7850	4.4995
Right lobe volume (Formula 2)	0.8800	1.0430	1.2225	1.6450	2.1600	3.4640	4.1215
Left lobe volume (Formula 1)	0.6640	0.7610	1.1825	1.8850	2.3700	3.3690	3.7450
Left lobe volume (Formula 2)	0.6075	0.7010	1.0800	1.7250	2.1700	3.0850	3.4310
Total thyroid volume (Formula 1)	1.7345	1.9890	2.7175	3.6300	4.5875	7.4730	7.9000
Total thyroid volume (Formula 2)	1.5915	1.8260	2.4925	3.3250	4.2000	6.8460	7.2320
**11–14 years (*n* = 34)**							
Right lobe volume (Formula 1)	1.0975	1.4500	2.1475	2.9850	3.9450	4.8800	5.3125
Right lobe volume (Formula 2)	1.0100	1.3300	1.9675	2.7300	3.6150	4.4700	4.8700
Left lobe volume (Formula 1)	1.1425	1.3050	1.7975	2.8550	3.6250	4.3450	5.0250
Left lobe volume (Formula 2)	1.0525	1.1950	1.6500	2.6150	3.3225	3.9800	4.6000
Total thyroid volume (Formula 1)	2.4675	2.8500	3.7700	5.9100	7.3375	8.8600	10.2625
Total thyroid volume (Formula 2)	2.2700	2.6100	3.4575	5.4050	6.7225	8.1200	9.3950
**15–18 years (*n* = 34)**							
Right lobe volume (Formula 1)	1.9950	2.2450	2.5500	3.2450	4.3075	5.1000	5.5950
Right lobe volume (Formula 2)	1.8275	2.0550	2.3400	2.9750	3.9450	4.6700	5.1275
Left lobe volume (Formula 1)	1.8200	2.0450	2.4300	3.1500	3.6350	4.0450	5.0675
Left lobe volume (Formula 2)	1.6625	1.8750	2.2225	2.8850	3.3325	3.7050	4.6425
Total thyroid volume (Formula 1)	3.9350	4.3800	4.8900	6.6200	8.1025	8.8050	10.2600
Total thyroid volume (Formula 2)	3.6025	4.0150	4.4800	6.0700	7.4250	8.0650	9.3975

Note: Values are in milliliters (mL). Percentiles calculated for each age group using two volume formulas (Formula 1: correction factor 0.523; Formula 2: correction factor 0.479).

**Table 3:** Significant correlation coefficients between total thyroid volume and other numerical variables.

Variable	Total Sample	4–10 Years	11–14 Years	15–18 Years
Age	r = 0.577, *p* < 0.001 *	ρ = 0.560, *p* = 0.001 *	ρ = 0.357, *p* = 0.038 *	n.s.
Height	r = 0.759, *p* < 0.001 *	ρ = 0.811, *p* < 0.001 *	ρ = 0.740, *p* < 0.001 *	ρ = 0.462, *p* = 0.006 *; ρ = 0.461, *p* = 0.006 *
Weight	r = 0.785, *p* < 0.001 *	ρ = 0.738, *p* < 0.001 *	ρ = 0.769, *p* < 0.001 *	ρ = 0.561, *p* = 0.001 *; ρ = 0.559, *p* = 0.001 *
Body mass index	r = 0.607, *p* < 0.001 *	ρ = 0.514, *p* < 0.001 *	ρ = 0.707, *p* < 0.001 *	ρ = 0.451, *p* = 0.007 *; ρ = 0.449, *p* = 0.008 *
Body surface area	r = 0.811, *p* < 0.001 *	ρ = 0.788, *p* < 0.001 *	ρ = 0.790, *p* < 0.001 *	ρ = 0.586, *p* = 0.001 *; ρ = 0.584, *p* = 0.001 *
Birthweight	r = 0.316, *p* = 0.002 *	n.s.	ρ = 0.370, *p* = 0.040 *	ρ = 0.360, *p* = 0.040 *; ρ = 0.359, *p* = 0.040 *
Number of comorbidities	r = 0.198, *p* = 0.049 *; r = 0.198, *p* = 0.048 *	n.s.	n.s.	n.s.
Calcitonin	r = −0.214, *p* = 0.032 *; r = −0.214, *p* = 0.033 *	n.s.	ρ = −0.364, *p* = 0.034 *	n.s.
Parathormone	n.s.	ρ = 0.460, *p* = 0.008 *	n.s.	ρ = −0.537, *p* = 0.001 *; ρ = −0.536, *p* = 0.001 *
Serum creatinine	r = 0.593, *p* < 0.001 *	ρ = 0.475, *p* = 0.006 *	ρ = 0.507, *p* = 0.002 *	ρ = 0.384, *p* = 0.025 *

Note: If both formulas yielded the same value, only one is reported. Otherwise, the first value refers to Formula 1 and the second to Formula 2. ρ—Spearman correlation coefficient; n.s.—not significant; r—Pearson correlation coefficient; *p*—Statistical significance; * Statistically significant (*p* < 0.05).

**Table 4:** Results of the final step of multiple linear regression analysis and significant predictors of the total thyroid volume.

Variable	Formula 1 (*n* = 96)			Formula 2 (*n* = 96)		
	B	95% CI	*p*	B	95% CI	*p*
Constant	−2.388	−3.910; −0.865	0.002 *	−2.184	−3.578; −0.790	0.002 *
Age	0.152	0.042; 0.261	0.007 *	0.139	0.039; 0.240	0.007 *
Body mass index	0.147	0.089; 0.205	<0.001 *	0.135	0.082; 0.187	<0.001 *
Serum creatinine	0.049	0.019; 0.079	0.002 *	0.045	0.017; 0.072	0.002 *
R^2^; F (*p*)	0.551; 37.592 (<0.001 *)			0.551; 37.611 (<0.001 *)		

Abbreviations: B—Unstandardized coefficient; CI—Confidence interval; *p*—Statistical significance; * Statistically significant (*p* < 0.05). Variables entered in the first step of the multiple linear regression analysis included: age, gender, body mass index, number of comorbidities, thyroid-stimulating hormone, free thyroxine, free triiodothyronine, calcitonin, parathormone, and creatinine.

## Data Availability

The data that support the findings of this study are available upon request from the corresponding author.
